# P-293. A Qualitative Exploration of the Acceptability of Long-Acting PrEP among Haitians/Haitian Americans and Community Leaders

**DOI:** 10.1093/ofid/ofaf695.514

**Published:** 2026-01-11

**Authors:** Candice A Sternberg, Claudine Berthold, Maika Beauvoir, Micaelle Titus, Morgan Philbin, Sannisha K Dale, Maria L Alcaide

**Affiliations:** University of Miami, Miami, FL; University of Miami, Miami, FL; University of Miami, Miami, FL; Community Health and Empowerment Network, North Miami, Florida; University of California, San Francisco, San Francisco, California; University of Miami, Miami, FL; Division of Infectious Diseases, Department of Medicine, University of Miami Miller School of Medicine, Miami, FL

## Abstract

**Background:**

Pre-exposure prophylaxis (PrEP) to prevent HIV, is effective and widely available in oral form, and increasingly in long-acting injectable (LAI) form, but there are disparities in use. Haitians and Haitian Americans in South Florida are disproportionately affected by HIV and have low uptake of HIV prevention modalities due to factors like stigma, low education, and access. Despite LAI PrEP (Cabotegravir) being approved in 2021, the use is low, and little is known about how this form compares to other existing modalities. This study provides insights on barriers to LAI PrEP among Haitians and Haitian Americans.

**Methods:**

We conducted semi-structured qualitative interviews in Miami, Florida, with 15 leaders in the Haitian community (e.g., nurse practitioners, pharmacists, psychologists, etc.) and 15 Haitian community members. Interviews occurred from August – November 2024 in partnership with the Community Health and Empowerment Network.

**Results:**

We interviewed 21 women and 9 men with an average age of 39 years. About 93% of community members were aware of PrEP and most preferred the LAI form. Three primary themes emerged: 1) conflicting views among interviewees about the role of stigma. Community leaders believed that stigma would strongly limit individuals’ willingness to take LAI PrEP, however, community members expressed autonomy in health decisions and prioritized health over social judgment; 2) community members expressed a preference for LAI PrEP over the oral form; 3) community members expressed primary barriers were access and education rather than medical mistrust and/or stigma.

**Conclusion:**

There are discrepancies in levels of stigma that may be influencing the use of LAI PrEP between community members and leaders in the Haitian community. Community members preferred the LAI PrEP and emphasized practical challenges over social concerns. To increase LAI PrEP uptake among Haitians, the approach should be multi-pronged and focused on addressing access and education gaps.

**Disclosures:**

Maria L. Alcaide, MD, Gilead: Advisor/Consultant

